# Capillary-driven surface-enhanced Raman scattering (SERS)-based microfluidic chip for abrin detection

**DOI:** 10.1186/1556-276X-9-138

**Published:** 2014-03-24

**Authors:** Hao Yang, Min Deng, Shan Ga, Shouhui Chen, Lin Kang, Junhong Wang, Wenwen Xin, Tao Zhang, Zherong You, Yuan An, Jinglin Wang, Daxiang Cui

**Affiliations:** 1 State Key Laboratory of Pathogen and Biosecurity, Beijing Institute of Microbiology and Epidemiology, No. 20 Dongda Street Fengtai District, Beijing 100071, People's Republic of China; 2 Institute of Nano Biomedicine and Engineering, Key Laboratory for Thin Film and Microfabrication Technology of the Ministry of Education, Research Institute of Micro/Nano Science and Technology, Shanghai Jiao Tong University, Dongchuan Road 800, 200240 Shanghai, People's Republic of China

**Keywords:** Capillary force, Microfluidic chip, SERS, Abrin, Phytotoxin

## Abstract

Herein, we firstly demonstrate the design and the proof-of-concept use of a capillary-driven surface-enhanced Raman scattering (SERS)-based microfluidic chip for abrin detection. The micropillar array substrate was etched and coated with a gold film by microelectromechanical systems (MEMS) process to integrate into a lateral flow test strip. The detection of abrin solutions of various concentrations was performed by the as-prepared microfluidic chip. It was shown that the correlation between the abrin concentration and SERS signal was found to be linear within the range of 0.1 ng/mL to 1 μg/mL with a limit of detection of 0.1 ng/mL. Our microfluidic chip design enhanced the operability of SERS-based immunodiagnostic techniques, significantly reducing the complication and cost of preparation as compared to previous SERS-based works. Meanwhile, this design proved the superiority to conventional lateral flow test strips in respect of both sensitivity and quantitation and showed great potential in the diagnosis and treatment for abrin poisoning as well as on-site screening of abrin-spiked materials.

## Background

Recently, ricin has caught the public's attention by the toxin-tainted letters sent to US President Barack Obama, Mississippi Senator Roger Wicker, and a Mississippi justice official, while abrin, its 70 times more toxic analogue, is less known to the general public. Abrin and ricin are toxic proteins with similar structure and properties, both of which are classified as category B select agents by the US Health and Human Services [1]. Compared with ricin, abrin is much more poisonous with an estimated human fatal dose of 0.1~1.0 μg/kg [2]. Although there are reported deaths on account of intentional poisoning, most cases occur in children by unintentional ingestion [3]. After ingestion, the major symptoms of abrin poisoning may occur in less than 6 h, and the deaths in children dying of ingestion of one or more abrin seeds have been documented in literature [4]. Therefore, a fast, readily available confirmatory testing will greatly facilitate the timely diagnosis and treatment for abrin poisoning.

Surface-enhanced Raman scattering (SERS) is a surface-sensitive technique that provides a highly enhanced Raman signal from Raman-active molecules that have been adsorbed onto rough metal surfaces. The reported surface enhancement factor ranges from 10^3^ to 10^15^, which means that the technique may detect proper analytes at a single molecule level [5-8]. There are two effects, chemical and electromagnetic, attributing to the enhancement. The former involves the formation of a charge-transfer state between the metal surface and adsorbate, contributing 1 to 2 orders of magnitude to the overall enhancement, while the latter is the dominant effect, arising from the collective oscillation of conduction electrons due to the irradiation of a metal by light [8]. Besides high sensitivity, the Raman scatter possesses 10~100 times narrower bands than those of fluorescence and excellent anti-photobleaching properties, which avail to reduce undesirable spectral overlap and provide long and stable signal readout [9]. So far, there have been many different SERS-based analytical techniques that have been developed for cancer markers, infectious diseases, pH sensing, etc. [8-15]. These techniques unleash tremendous potential for ultrasensitive biomedical analysis. However, it still remains a great challenge to reduce the overall cost while maintaining the advantages of sensitivity, because most SERS-based detection systems are strongly dependent on the relatively expensive process of microelectromechanical systems (MEMS), especially sputtering of a noble metal layer.

**Figure 1:**
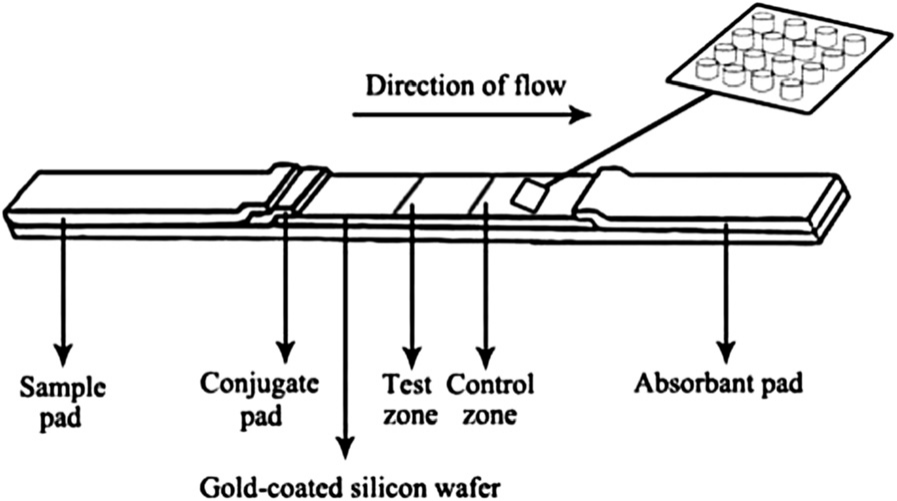
The schematic view of capillary-driven SERS microfluidic chip.

Herein, we introduce a proof-of-concept use of the capillary-driven SERS-based microfluidic chip for abrin detection (Figure 1). A micropillar array was fabricated by MEMS process on silicon wafer and sputtered with noble metal. After proper hydrophilic modification, anti-abrin polyclonal antibodies and secondary antibodies were immobilized on different places of the micropillar array as the detection zone and control zone. The sample liquid dissolved the external anti-abrin SERS probes in the conjugate pad and reacted with them and then was driven through the whole micropillar array by capillary action. The detection signal was provided by the external SERS probes captured on the detection and control zones. This proof-of-concept design combined the advantages of SERS-based detection and previous capillary action-driven chip, providing a novel and feasible solution for the application of SERS-based point-of-care test (POCT).

## Methods

All animal experiments (No. SYXK2007-0025) were approved by the Institutional Animal Care and Use Committee of Shanghai Jiao Tong University.

### Extraction of natural abrin

Natural abrin was extracted according to the previous method with slight modifications [16]. Briefly, the decorticated seeds of *Abrus precatorius* (approximately 100 g) were soaked in 200 mL of 0.01 M phosphate buffer solution (PBS) at pH 7.4 and 4°C for 24 h. After thorough homogenization, the puree was centrifuged at 10,000*g* for 30 min. Then, the aqueous layer was saturated with ammonium sulfate (95% *w*/*v*) and centrifuged at 10,000*g* for 30 min. The precipitate was dissolved in 100 mL of 0.01 M PBS and applied to a 1.5 × 10 cm Gal-agarose column (EY Laboratories Inc., San Mateo, CA, USA). The bound abrin was eluted with 0.125 M d-galactose solution. The collected fractions were dialyzed and applied to a Sephacryl S-100 prepacked column (GE Healthcare Bio-Sciences Corp, Piscataway, NJ, USA) equilibrated in PBS. The as-prepared abrin was analyzed by 15% sodium dodecyl sulfate polyacrylamide gel electrophoresis (SDS-PAGE).

### Preparation of anti-abrin polyclonal antibodies

The purified abrin was inactivated by formalin and used to hyperimmunize a rabbit, and 0.5 mL of abrin toxoid (80 mg/mL) was mixed with an equal volume of Freund's complete adjuvant and injected subcutaneously to the rabbit. Seven days later, immunization was carried out four times including one booster immunization with the mixture of the abrin toxoid and Freund's incomplete adjuvant as well as three injections with the toxoid at weekly intervals. Ten days after the final injection, the immunized blood was collected by jugular puncture, and the serum was separated for subsequent purification of anti-abrin polyclonal antibodies with rProtein A Sepharose Fast Flow (GE Healthcare Bio-Sciences Corp., Piscataway, NJ, USA). The antibody titers were evaluated by enzyme-linked immunosorbent assay (ELISA).

### Preparation of external SERS probes

The external SERS probes were prepared according to a published method [6]. DTNB (5,5′-dithiobis (2-nitrobenzoic acid), Sigma-Aldrich Co. LLC, St. Louis, MO, USA) was used as the Raman-active tag. One milliliter of purified anti-abrin polyclonal antibodies (approximately 75 mg/mL in 0.01 M PBS) was dropwise added to 1 mL of 20-nm colloidal gold solution (Sigma-Aldrich Co. LLC) under stirring. After 1 h of incubation at 4°C, the antibody-coated colloidal gold was separated by centrifugation at 12,000*g* for 1 h. Bovine serum albumin (BSA) was used to block the unmodified colloidal gold at a final concentration of 0.5% (*w*/*v*). The labeled colloidal gold was centrifuged at 12,000*g* for 1 h and resuspended in 1 mL 0.01 M PBS solution. Twenty microliters of DTNB solution (1 mM in 0.01 M PBS) was added to the gold solution and incubated at 4°C for 1 h. The resultant SERS probes were centrifuged again at 12,000*g* for 1 h and then resuspended in 0.01 M PBS for later use.

### Fabrication and surface modification of gold-coated silicon wafer

**Figure 2:**
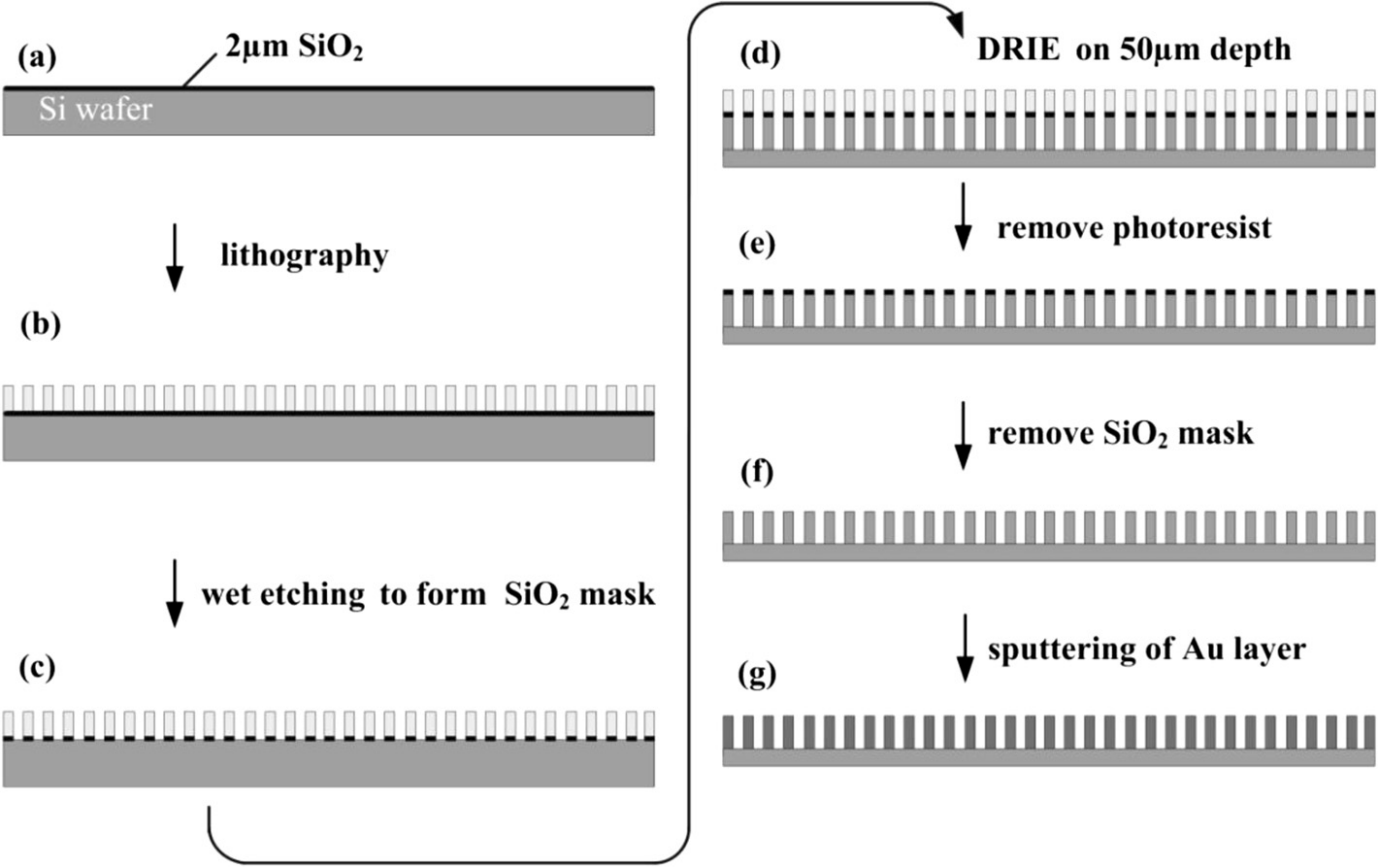
**The fabrication process for the capillary-driven SERS-based microfluidic chip. (a)** SiO_2_ film(2 μm) was grown onto a Si wafer using wet oxidation. **(b)** Lithography. **(c)** SiO_2_ was wet-etched by BHF. **(d)** Si wafer was dry-etched by DRIE. **(e, f)** Removal of photoresist and SiO_2_ mask. **(g)** Au film (200 nm) was deposited on the pattern.

The gold-coated silicon wafer was fabricated by MEMS technique. The process was shown in Figure 2. Firstly, a 2-μm-thick layer of SiO_2_ was grown onto a 3-in. Si wafer (Mouser Ltd., Hefei, China) using wet oxidation in a thermal furnace (TS-6304, Tempres Ltd., Vaasen, The Netherlands). Then, a photoresist (AZ 4562, Micro Chemicals Ltd., Japan) was spin-coated at 3,000 rpm to a thickness of approximately 20 μm and soft-baked for 90 min at 80°C. The layer was patterned subsequently by photolithography. The buffered hydrofluoric acid (BHF, composition of BHF solution for SiO_2_ etching: HF 84 mL, NH_4_F 339 g, H_2_O_5_ 10 mL; etching condition: 45°C, pH 3) was used to etch SiO_2_ uncovered by the photoresist. Afterwards, the microchips were etched into Si to a depth of 50 μm using deep reactive ion etching (DRIE, AMS-200SE, Annecy Cedex Ltd., France), and the micro silicon cylindrical array formed. The photoresist and SiO_2_ mask were removed by acetone (Great Fortune, Zibo Ltd, China) and DRIE, respectively. The as-prepared chips were cut into strips (1 × 4 mm) using a laser scribing apparatus (WL-9030, Titan Ltd., USA). After being cleaned with the reactive ion etcher (Nextral-100, Alcatel Ltd., France) at 30 W and 1.2 Torr for 45 s, the chips were then incubated in a solution of acetone for 20 min, rinsed with deionized water, and dried under an N_2_ stream. The deposition of gold film (200 nm) on the chip was carried out with the sputtering system (ZT-550, L-H Ltd., Germany). After being soaked in piranha solution (H_2_SO_4_/30% H_2_O_2_ = 3:1) for 10 min, the gold-coated chips were cleaned with deionized water and rinsed in 1 mM of HS-C_2_H_4_-CONH-PEG-C_3_H_6_-COOH (Rapp Polymere GmbH Ltd., Tuebingen, Germany) solution for 4 h. Finally, the chips were cleaned with deionized water and dried at room temperature. Scanning electron microscopy (SEM) (JEOL Ltd. Tokyo, Japan) was used to explore the surface ultrastructure of the as-prepared chip. PBS was used to evaluate the flow rate of the sample solution on the chip.

### Assembly of capillary-driven chip

Anti-abrin polyclonal antibodies and goat anti-rabbit secondary antibodies (1 mg/mL) were dispensed on the gold-coated wafer with a Biodot XYZ3000 dispenser (Biodot Inc., Irvine, CA, USA) as test zone and control zone, respectively. After drying for 30 min, the wafer was blocked with PBS containing 1% BSA (*w*/*v*). The SERS probes were printed on a glass fiber filter as conjugate pad and dried at room temperature. The absorbent pad, conjugate pad, and sample pad were cut into strips of 1 mm in width with a guillotine cutter and overlapped on the laminating card with the dried wafer as shown in Figure 1.

### SERS signal measurement

The purified abrin was diluted with a series of concentrations from 0.1 ng/mL to 10 mg/mL in 0.01 M PBS solution. Fifty microliters of the diluted toxin solution was added to the sample pad, and the SERS signal was read out with i-Raman-785S (B&W TEK Inc., Newark, DE, USA) after 5 min. The intensity of the peak at approximately 1,330 cm^-1^ was used to quantify the abrin in PBS solution.

## Results and discussion

### Characterization of natural abrin and anti-abrin antibody

**Figure 3:**
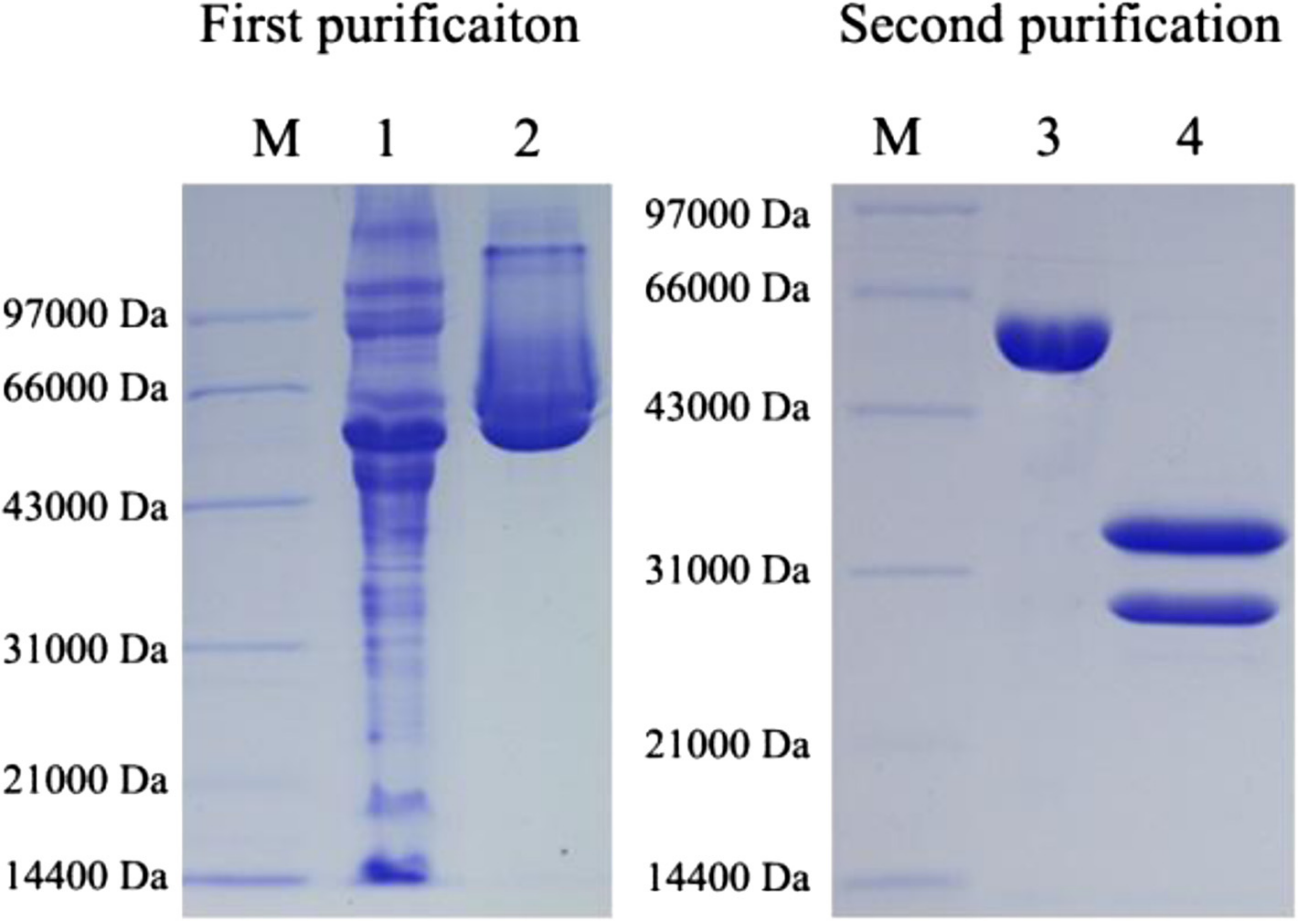
**SDS-PAGE analysis of purified abrin.** M, protein marker; 1, raw extract; 2, purified abrin by the first step; 3, purified abrin by the second step under nonreducing condition; 4, purified abrin by the second step under reducing condition.

Abrin consists of two subunits which are linked by a disulfide bond between Cys247 of the A subunit and Cys8 of the B subunit [2]. Their molecular weights are approximately 30 and 35 kDa, respectively. The A subunit inactivates ribosomes and inhibits protein synthesis, while the B subunit shows the activity of lectin that facilitates the entrance into the cell. The extraction of natural abrin with high purity is the key in production of polyclonal antibody, which determines the quality of induced antibody. However, the process of the purification of abrin from seeds of *A. precatorius* was complicated due to the existence of abundant agglutinin that possesses nearly identical galactose-binding properties as abrin. Given their differences in galactose-binding avidity and molecular mass between the abrin and agglutinin, a two-step purification was exploited to separate abrin from raw extracts (Figure 3). As shown in Figure 2, the purified abrin in the final step could be broken into two subunits under reducing condition, and the sizes of bands were in accordance with their theoretical molecular weight. In addition, the purity was over 95% by Quantity One software analysis (Bio-Rad Laboratories Inc., Hercules, CA, USA). After being inactivated with formalin, the abrin toxoid was used to produce polyclonal antibody. In this experiment, the as-prepared antibody could yield a positive result by ELISA under 100,000-fold dilution, which reflected the good immunogenicity of the abrin toxoid and good affinity of the antibodies.

### Characterization of microfluidic chip

**Figure 4:**
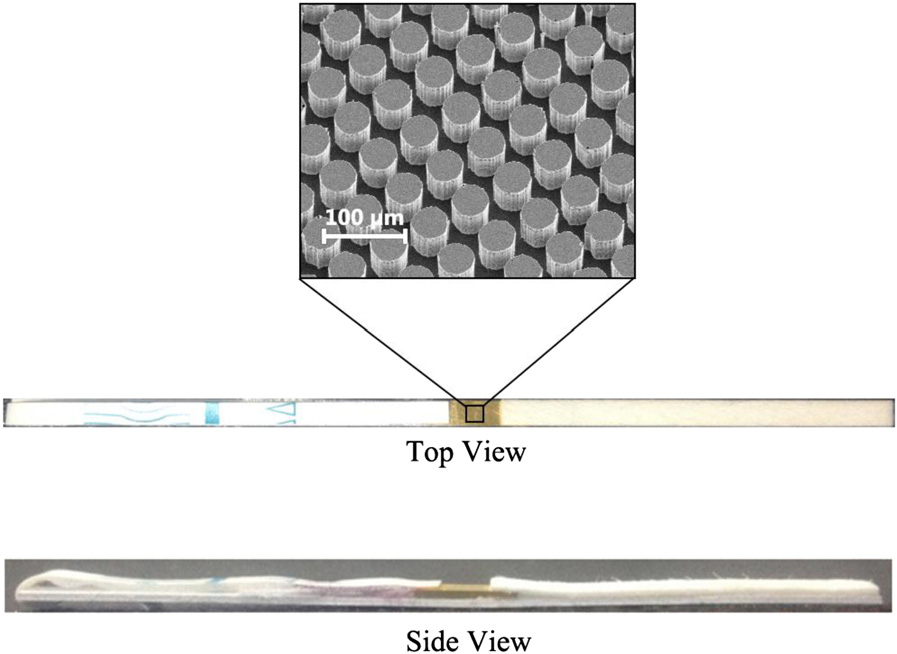
Characterization of capillary-driven SERS microfluidic chip.

The assembled microchip is shown in Figure 4. From the appearance, it resembled a traditional lateral flow (LF) test strip except for its width (1 mm) and gold-coated substrate. The SEM image showed the micropillar array on the chip. The micropillars were about 50 μm high and had a diameter of 35 μm and a center-to-center distance of 90 μm. The flow rate of PBS was about 4 mm/s on the chip. In this experiment, the design of microchip referred to the microstructure of micropost array of 4castchip^®^ developed by Åmic AB [17,18]. It is important to note that the LF strip is one of the most successful commercial POCT products. So far, there was no available commercial POCT product that overmatches the lateral flow test strip in cost and universality of application. However, the main weaknesses of the colloidal gold or latex-based traditional LF test trip are sensitivity and quantitation as a result of the intrinsic property of the cellulose membrane [19-22]. Particularly, it is only the superficial colorimetric signal that could be used for quantitation, while the deep signal in the membrane is lost. The planar structure of 4castchip^®^ addressed the problem well and retained the capability of capillary-driven force. However, it is obvious that the cost for sputtering noble metal will be high if this structure is wholly introduced into the SERS-based chip. In fact, except for cellulose membrane, other components of the traditional LF strip function well and cost little in practical application. Therefore, only the cellulose membrane was replaced by the gold-coated micropillar array substrate in our design strategy. This strategy has several advantages as follows: First of all, it economizes the consumption of gold-coated substrate and facilitates the homogeneous batch processing. Second, it is a mature technique in practice to process the sample pad, conjugate pad, and absorbent pad, which works well and does not need further optimization. Third, wide center-to-center distance guarantees a good passing ability, avoiding the blocking of possible residual coarse materials passing through sample pad in samples. Last but not the least, this design, which decreased the width of flow path in conventional LF test strips from 4 to 1 mm, facilitates the enriching of analytes on the surface of the capture zone, improving the sensitivity under the condition of high flow rate. In other words, this strategy reduced not only the complication of fabrication, but also the overall cost.

### Analysis of abrin-spiked sample

**Figure 5:**
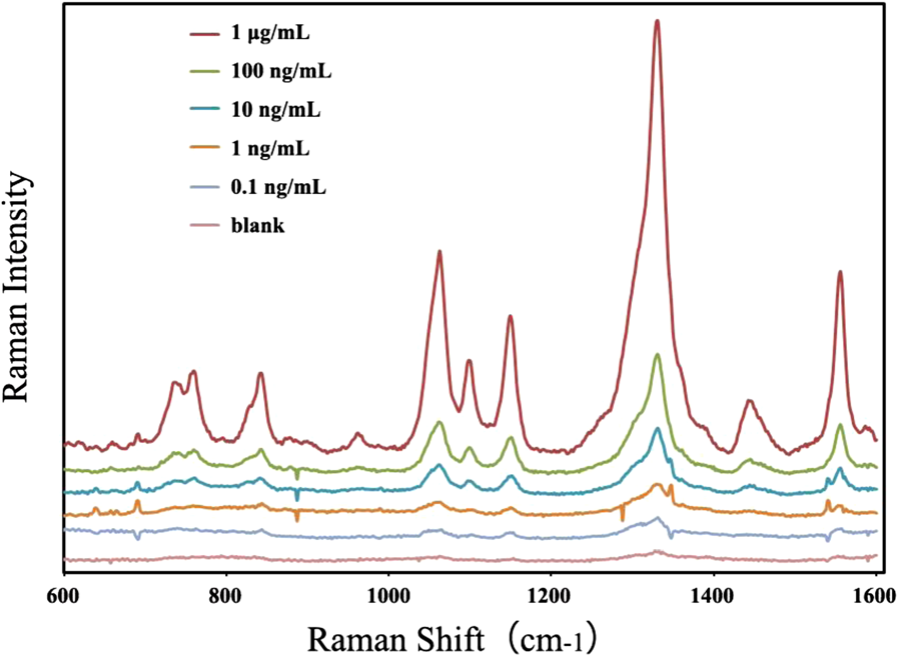
SERS spectra of the abrin-spiked sample at different concentrations.

**Figure 6:**
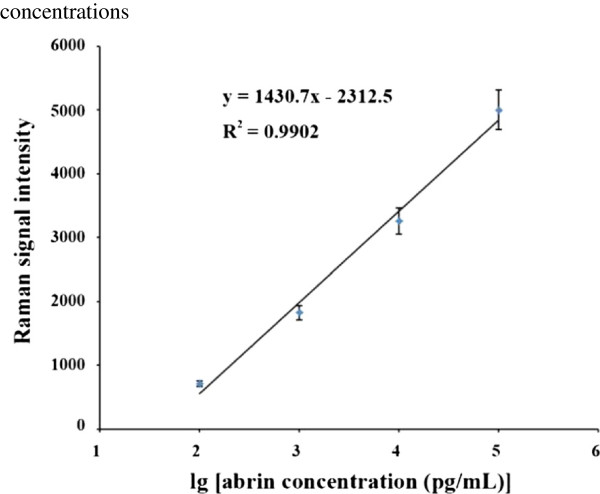
Dose–response curve for the abrin-spiked sample at different concentrations.

Figure 5 shows the SERS spectra of the abrin-spiked sample at various concentrations. The intensity of the peak at approximate 1,330 cm^-1^ was proportional to the concentration of abrin in the PBS solution. The concentration of abrin ranged from 0.1 ng/mL to 1 μg/mL. The characteristic peak under 0.1 ng/mL became difficult to distinguish from that of the blank sample, indicating the limit of detection (LOD). Because of the absence of the washing step, some SERS probes remained on the gold-coated substrate, resulting in a weak nonspecific binding peak at approximate 1,330 cm^-1^. Figure 6 shows the dose-response curve calculated by averaging the readout at three different locations of each concentration from 0.1 to 100 ng/mL. The linear regression equation was *y* =1,430.7*x* - 2,312.5 and the correlation coefficient (*R*^2^) was 0.9902. The LOD of this capillary-driven SERS-based microfluidic chip was 0.1 ng/mL.

As previously mentioned, SERS-based techniques showed many potential advantages including high sensitivity, narrow bandwidths, and photobleaching resistance. It still remains a challenge to develop a SERS-based immunodiagnostic technique of both low cost and good operability. Some pioneering researchers have published their works focusing on the ultrasensitivity from the level of picograms per milliliter to femtograms per milliliter [6,8,9,11,14,23-28]. Compared with their work, our design strategy emphasized the operability of SERS-based technique. In other words, this strategy is aimed at not just a comparative LOD, but a balanced solution between the complication of new techniques and the universality of traditional ones.

## Conclusions

We have designed and demonstrated the proof-of-concept use of our capillary-driven SERS-based microfluidic chip for abrin detection. The microfluidic chip was fabricated by MEMS process. The combination of the traditional LF test strip with capillary-driven gold-coated substrate results in the enhancement of sensitivity as well as the reduction of cost for SERS-based immunodiagnostic techniques. In this work, a calibration curve was obtained to detect the concentration of abrin in the range from 0.1 ng/mL to 1 μg/mL, which is superior to the traditional LF test strip for the same purpose in respect of both sensitivity and quantitation [21]. What is critically important is the operability of our design strategy, that is, the performance of traditional LF test strips is improved without excessive increase in complication and cost of fabrication. In addition, this SERS-based microfluidic chip can be further developed and applied to other on-demand and point-of-care detection for a substance of interest.

## Competing interests

The authors have declared that no competing interest exists.

## Authors’ contributions

HY carried out antibody preparation and SERS experiments. MD finished the microfabrication of the micropillary chip. SG finished the surface modification of the micropillary chip. SHC finished the antibody conjugation with the surface of the chip. LK and JW finished the characterization of the chip. WX, TZ, and ZY finished the result analysis. HY and YA finished the draft. JW and DC finished the experiment design and manuscript revision. All authors of this paper have read and approved the final manuscript.

## References

[B1] Felder E Mossbrugger I Lange M Wolfel R Simultaneous detection of ricin and abrin DNA by real-time PCR (qPCR) Toxins 2012 9 633 642 10.3390/toxins4090633 23105972 PMC3475220

[B2] Dickers KJ Bradberry SM Rice P Griffiths GD Vale JA Abrin poisoning Toxicol Rev 2003 9 137 142 10.2165/00139709-200322030-00002 15181663

[B3] Jang DH Hoffman RS Nelson LS Attempted suicide, by mail order: Abrus precatorius J Med Toxicol 2010 9 427 430 10.1007/s13181-010-0099-1 20563676 PMC3550472

[B4] Balali-Mood M Moshiri M Etemad L Medical aspects of bio-terrorism Toxicon 2013 9 131 142 23339855 10.1016/j.toxicon.2013.01.005

[B5] Yang D-P Chen S Huang P Wang X Jiang W Pandoli O Cui D Bacteria-template synthesized silver microspheres with hollow and porous structures as excellent SERS substrate Green Chem 2010 9 2038 2042 10.1039/c0gc00431f

[B6] Lin CC Yang YM Chen YF Yang TS Chang HC A new protein A assay based on Raman reporter labeled immunogold nanoparticles Biosens Bioelectron 2008 9 178 183 18468881 10.1016/j.bios.2008.03.035

[B7] Nie S Emory SR Probing single molecules and single nanoparticles by surface-enhanced Raman scattering Science 1997 9 1102 1106 10.1126/science.275.5303.1102 9027306

[B8] Porter MD Lipert RJ Siperko LM Wang G Narayanan R SERS as a bioassay platform: fundamentals, design, and applications Chem Soc Rev 2008 9 1001 1011 10.1039/b708461g 18443685

[B9] Grubisha DS Lipert RJ Park HY Driskell J Porter MD Femtomolar detection of prostate-specific antigen: an immunoassay based on surface-enhanced Raman scattering and immunogold labels Anal Chem 2003 9 5936 5943 10.1021/ac034356f 14588035

[B10] Zong S Wang Z Chen H Hu G Liu M Chen P Cui Y Colorimetry and SERS dual-mode detection of telomerase activity: combining rapid screening with high sensitivity Nanoscale 2014 9 1808 1816 10.1039/c3nr04942f 24356868

[B11] Wu P Gao Y Lu Y Zhang H Cai C High specific detection and near-infrared photothermal therapy of lung cancer cells with high SERS active aptamer-silver-gold shell-core nanostructures Analyst 2013 9 6501 6510 10.1039/c3an01375h 24040647

[B12] Zhang P Zhang R Gao M Zhang X Novel nitrocellulose membrane substrate for efficient analysis of circulating tumor cells coupled with surface-enhanced Raman scattering imaging ACS Appl Mater Interfaces 2014 9 370 376 10.1021/am404406c 24325273

[B13] Yuen C Liu Q Optimization of Fe3O4@Ag nanoshells in magnetic field-enriched surface-enhanced resonance Raman scattering for malaria diagnosis Analyst 2013 9 6494 6500 10.1039/c3an00872j 24049766

[B14] Lee S Chon H Lee J Ko J Chung BH Lim DW Choo J Rapid and sensitive phenotypic marker detection on breast cancer cells using surface-enhanced Raman scattering (SERS) imaging Biosens Bioelectron 2014 9 238 243 23973735 10.1016/j.bios.2013.07.063

[B15] Kong KV Dinish US Lau WK Olivo M Sensitive SERS-pH sensing in biological media using metal carbonyl functionalized planar substrates Biosens Bioelectron 2014 9 135 140 24269755 10.1016/j.bios.2013.10.052

[B16] Tang J Xie J Shao N Yan Y The DNA aptamers that specifically recognize ricin toxin are selected by two in vitro selection methods Electrophoresis 2006 9 1303 1311 10.1002/elps.200500489 16518777

[B17] Jonsson C Aronsson M Rundstrom G Pettersson C Mendel-Hartvig I Bakker J Martinsson E Liedberg B MacCraith B Ohman O Melin J Silane-dextran chemistry on lateral flow polymer chips for immunoassays Lab Chip 2008 9 1191 1197 10.1039/b800297e 18584097

[B18] Melin J Rundstrom G Peterson C Bakker J MacCraith BD Read M Ohman O Jonsson C A multiplexed point-of-care assay for C-reactive protein and N-terminal pro-brain natriuretic peptide Anal Biochem 2011 9 7 13 10.1016/j.ab.2010.09.034 20875778

[B19] Posthuma-Trumpie GA Korf J van Amerongen A Lateral flow (immuno)assay: its strengths, weaknesses, opportunities and threats. A literature survey Anal Bioanal Chem 2009 9 569 582 10.1007/s00216-008-2287-2 18696055

[B20] Yang H Li D He R Guo Q Wang K Zhang X Huang P Cui D A novel quantum dots-based point of care test for syphilis Nanoscale Res Lett 2010 9 875 881 10.1007/s11671-010-9578-1 20672123 PMC2893857

[B21] Gao S Nie C Wang J Kang L Zhou Y Wang JL Colloidal gold-based immunochromatographic test strip for rapid detection of abrin in food samples J Food Protect 2012 9 112 117 10.4315/0362-028X.JFP-11-252 22221362

[B22] Yang H Guo Q He R Li D Zhang X Bao C Hu H Cui D A quick and parallel analytical method based on quantum dots labeling for ToRCH-related antibodies Nanoscale Res Lett 2009 9 1469 1474 10.1007/s11671-009-9422-7 20652102 PMC2894333

[B23] Tian S Zhou Q Gu Z Gu X Zheng J Fabrication of a bowl-shaped silver cavity substrate for SERS-based immunoassay Analyst 2013 9 2604 2612 10.1039/c3an36792d 23476921

[B24] Chon H Lee S Son SW Oh CH Choo J Highly sensitive immunoassay of lung cancer marker carcinoembryonic antigen using surface-enhanced Raman scattering of hollow gold nanospheres Anal Chem 2009 9 3029 3034 10.1021/ac802722c 19301845

[B25] Wu L Wang Z Zong S Huang Z Zhang P Cui Y A SERS-based immunoassay with highly increased sensitivity using gold/silver core-shell nanorods Biosens Bioelectron 2012 9 94 99 22647534 10.1016/j.bios.2012.05.005

[B26] Xu S Ji X Xu W Zhao B Dou X Bai Y Ozaki Y Surface-enhanced Raman scattering studies on immunoassay J Biomed Optic 2005 9 031112 10.1117/1.1915487 16229637

[B27] Yoo JH Han HS Lee C Yoo KP Kang T Surface-enhanced Raman scattering-based detection of molecules in an aqueous solution via lipid-modified gold nanorods J Nanosci Nanotechnol 2013 9 7239 7244 10.1166/jnn.2013.8087 24245236

[B28] Pekdemir ME Erturkan D Kulah H Boyaci IH Ozgen C Tamer U Ultrasensitive and selective homogeneous sandwich immunoassay detection by surface enhanced Raman scattering (SERS) Analyst 2012 9 4834 4840 10.1039/c2an35471c 22943047

